# Prevention of HIV and Other Sexually Transmitted Infections by Geofencing and Contextualized Messages With a Gamified App, UBESAFE: Design and Creation Study

**DOI:** 10.2196/14568

**Published:** 2020-03-17

**Authors:** Felipe Besoain, Antoni Perez-Navarro, Constanza Jacques Aviñó, Joan A Caylà, Nicolas A Barriga, Patricia Garcia de Olalla

**Affiliations:** 1 School of Videogames Development and Virtual Reality Engineering Faculty of Engineering​ Universidad de Talca Campus Talca Chile; 2 Internet Interdisciplinary Institute Universitat Oberta de Catalunya Castelldefels Spain; 3 Faculty of Computer Sciences, Multimedia and Telecommunication Universitat Oberta de Catalunya Barcelona Spain; 4 Epidemiologic Service - Public Health Agency of Barcelona Plaça Lesseps 1 Barcelona Spain; 5 Foundation of the Tuberculosis Research Unit of Barcelona Barcelona Spain; 6 Biomedical Research Network on Epidemiology and Public Health (CIBEResp) Barcelona Spain

**Keywords:** human immunodeficiency virus, mobile apps, sexually transmitted infection, recreational games

## Abstract

**Background:**

Advances in the development of information and communication technologies have facilitated social and sexual interrelationships, thanks to the websites and apps created to this end. However, these resources can also encourage sexual contacts without appropriate preventive measures in relation to HIV and other sexually transmitted infections (STIs). How can users be helped to benefit from the advantages of these apps while keeping in mind those preventive measures?

**Objective:**

This study aimed to prevent STIs by helping users to remember preventive measures in the risky situations.

**Methods:**

We have used the design and creation methodology and have developed a software system. This system has two parts: an Android operating system app with emphasis on ubiquitous computing and gamification as well as a server with a webpage. First, a functional test with 5 men who have sex with men (MSM) allowed us to test the app with end users. In addition, a feasibility test with 4 MSM for a month allowed us to try the UBESAFE system with all its functionalities.

**Results:**

The main output is a system called UBESAFE that is addressed to MSM. The system has two main parts: (1) an app that sends preventive contextualized messages to users when they use a contact app or when they are near a point where sexual contacts are likely and (2) a server part that was managed by the public health agency of Barcelona (ASPB), which preserves the quality and pertinence of messages and places and offers instant help to users. To increase users’ adherence, UBESAFE uses a gamified system to engage users in the creation of preventive messages. Users increased the initial pool of messages by more than 100% (34/30) and created more than 56% (9/16) of places (named hot zones).

**Conclusions:**

The system helped MSM who used it to become conscious about HIV and other STIs. The system also helped the ASPB to stay in contact with MSM and to detect behaviors that could benefit from preventive measures. All functions were performed in a nonintrusive manner because users used the app privately. Furthermore, the system has shown how important it is to make users a part of the creation process as well as to develop apps that work by themselves and thus become useful to the users.

## Introduction

### Background

Currently, many health organizations work actively to decrease the number of HIV infections. Despite the major advances in the treatment of HIV, prevention of infection is still better than treatment [1]. In the recent years, the number of health campaigns and the number of locations where such health campaigns are implemented have grown substantially [2]. Nowadays, many different types of methods are available to disseminate information about health and prevention of diseases. Some of the main methods include publicity projects, outreach work with groups of individuals who may be at risk, the monitoring and control at a national level of items recognized as having a negative impact on health, programs at educational institutions, and the use of social media, to name a few [3]. Nevertheless, it is important to consider the impact of the information and communication technology in social relationships. Many studies show how the internet has been considered as a connection point to meet sexual partners [4,5].

However, with the introduction of smartphones and tablets to the market, information is increasingly omnipresent and, as a consequence, the access to apps and social networks is more ubiquitous [6,7]. Dating mobile apps are also modern tools to find sexual partners through the internet, considering aspects such as location, timing, and taste, among others. It has also been shown how dating apps impact HIV infection for many reasons: first, users can find sexual partners easily on the go; second, users maximize the likelihood to find a sexual partner because apps are free; and third, there are dating apps for heterosexual and gay and bisexual communities. People who use the dating apps and have these sexual conducts usually take more risks when they have a sexual encounter [8].

Researchers have examined the main tools that are currently used for preventing the spread of HIV [9]. These tools include Web-based and mobile apps, games, and social media, among others. The target of many of these campaigns and of several strategic plans to control HIV infections are men who have sex with men (MSM) because they are considered a high-risk group in most European and American countries. In this context, most efforts have been directed toward prevention through the use of apps that provide information when facing specific situations and behavior. It has also been suggested that researchers in public health should work with app developers to incorporate innovative elements, starting with interventions that reduce the risk and the associated behaviors, as well as that improve the inclusivity and interactivity of the apps [10].

Choi et al [11] showed how an app can help introduce healthier behaviors regarding HIV, although they found no concluding remarks about risk reduction. Alarcon et al [12] have shown that sending messages through apps helps to promote testing for HIV and other sexually transmitted infections (STIs). Biello et al [13] proposed a study to analyze an app that promotes the uptake of HIV testing and pre-exposure prophylaxis. Although this last app has a similar target to that proposed in this paper, it is an informative app that is focused on informing about how to get tested and where to get prophylaxis measures, but not on sending context-based messages as the one proposed in this paper. Chow et al [14] show how using geolocalized apps offer several opportunities in HIV prevention. However, as far as we know, this is the first time that context of the users is used to prevent risky behaviors in HIV, thanks to the characteristics and omnipresence of smartphones.

### This Work

This work addresses these drawbacks by developing an app to send preventive notifications to users when it detects situations such as the activation of a particular app (dating app) on their smartphone, or their proximity to areas with a high probability of intercourse. To increase adherence, the app uses gamification techniques. The development process has been performed in a co-design process with potential users (MSM) with a goal of developing a system that has value for users per se [15] and increases users’ adherence and preventive effect. This work is the continuation of work by Besoain et al [16] in which the use of mobile devices and their ubiquity was used to prevent STIs.

We based our design of this technological approach on the elaboration likelihood model that describes a framework of multiple processes in which communication variables (eg, channel or message) can change people’s attitudes and ultimately their behavior [17]. Several investigations in social psychology have consistently shown that the thoughts that people generate in response to social information are important predictors of their attitudes and behaviors [18]. Many of the studies on attitude change use specific communicative information (persuasive messages) to generate thoughts of different directionality. Therefore, messages have been used with arguments in a favorable or unfavorable direction (see for a review [19]). When a person receives arguments that are strong, this tends to generate thoughts in line with the information [20].

Therefore, in this app, participants received, and were asked to help the health community to generate, favorable arguments for healthy behavior, such as using condoms, with the aim of generating thoughts in this direction and positively impacting users’ future decisions. However, it is important to note that in this study, our objective was to develop the technology and test the main idea in general terms. More specific studies about the effects of the arguments for healthy behavior with this technology will be a part of future work.

This paper describes an app developed for HIV prevention called UBESAFE. To prevent users from feeling that the app has an overbearing monitoring effect, it has been designed in a way that allows them to actively participate in the creation of a tool to reinforce healthy behavior. Users download UBESAFE and configure it themselves. They can use the app in two main ways, to reinforce healthy behavior when (1) using contact apps that could be used for initiating sexual relationships (Grindr, ManHunt, etc) and (2) walking or passing by a geographical area (hotzone) where sexual activities could occur, for example, gay saunas and nightclubs. The users select the contact apps alone or the contact apps along with geographical areas that they want to include in UBESAFE, taking an active role in the process. When users engage in one of these activities (use a preselected contact app or move through a preselected hotzone), UBESAFE sends a health notification message. These HIV prevention messages are not designed to discourage sexual relationships, but rather to encourage users to make healthy decisions and increase the awareness of their sexual health (for example, do not forget to use condoms). The HIV prevention messages are written by MSM with the guidance of health professionals. Furthermore, to encourage an active role, users can write their own messages. In a gamification aspect of the app, users can earn points if they post messages. It is important to note that UBESAFE does not intend to stigmatize sex, but rather encourage awareness and healthy sex decisions to contribute to HIV prevention.

The paper is structured as follows: (1) the methods used to create the app are introduced, and the architecture and use cases of the app are shown; (2) the results, which are mainly the main features of the app and its utility as a preventive tool, are presented; and (3) our conclusions and future work in this area are described.

## Methods

### Overview

The method used in this research follows the *design and creation* approach to create a system with two parts: a management web server for health professionals; and an app for users, which is the main preventive element. The app uses ubiquitous computing concepts [21], localization services [22], and game mechanics [23,24] to prevent HIV infections and other STIs. This process has been divided in two steps: (1) a co-design methodology to enhance and address the functional requirements of the app by a group of potential users [15], and (2) a functional and a feasibility test has been performed to evaluate the app with a target group [25,26].

To design and develop the app, the following methodologies have been followed:

- The design process was performed with health experts and the target group. It is important to note that because the app will be a supervised app, both health experts and target group will be users of different parts of the app, and they both will pursue different goals. This co-design methodology plays a key role because we plan to create an app useful for both kinds of users, and it is important to create something with added value for every single user, but trying to avoid interference, from the user experience (UX) point of view, between the different goals. The co-design was performed through focus groups, surveys, and functional testing. The result is an incremental process that has gone through three previous apps:
  - Ubiapp used geofencing to recognize different hotspots (places with high probability of sexual encounter) and the use of a list of risky apps (dating apps) for delivering a health message [16]. In the development of this app, a study with 17 MSM helped obtain a pool of messages that were later used as the seed pool of messages of UBESAFE.
  - Ubinut launches health messages addressed to prevent obesity and allow the users to score them on a Likert scale [27].
  - Geonut has the same functionality as Ubinut, but it also incorporates geofencing to hotspots (in this case, places with restaurants, fast food courts, among others).

To test the app and choose the health or preventive messages, a test was performed in two steps. First, a functional test was performed with 5 MSM for 2 weeks with the aim of receiving feedback on the UX through a focus group. The number of the participants in the study was based on the open call for volunteers through the public health agency of Barcelona (ASPB); the volunteers were MSM HIV-negative. The number is considered sufficient because the objective of this study was to test technical aspects of the app in a real situation and also receive initial feedback from the user. The second study was a feasibility test that was then performed with 4 MSM for a month to try the UBESAFE system with all of its functionalities. These participants were the same as those from the first study, except for one participant who could not continue because of lack of time. The purpose of this initial test was to receive feedback on the UX through a focus group with MSM. The previous experiences with Ubiapp, Ubinut, and Geonut were taken into account to test the system.

**Figure 1:**
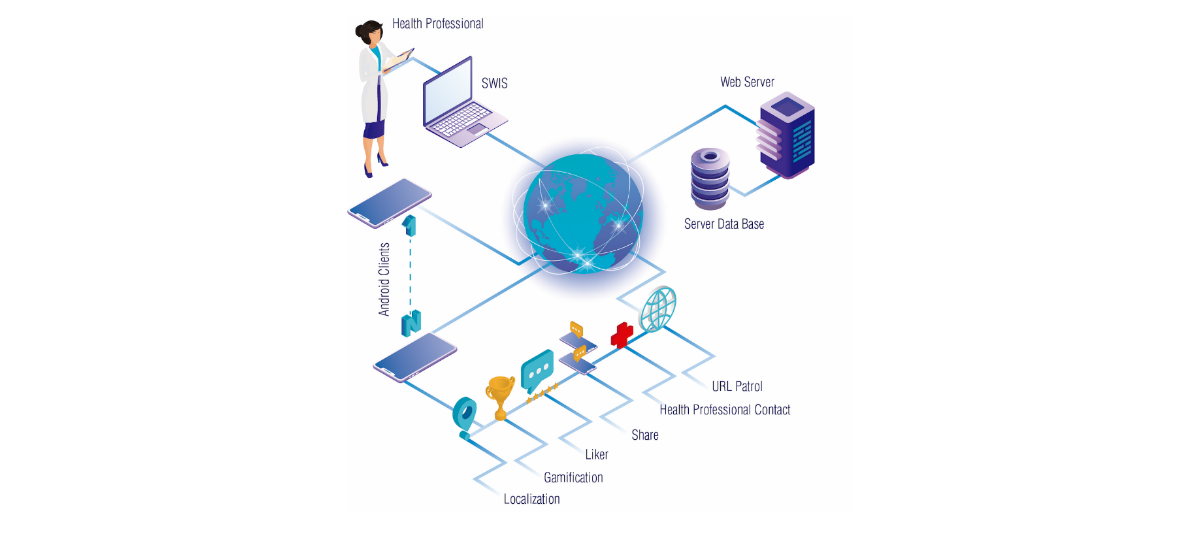
Architecture of solution and components of the UBESAFE system. This includes (1) simple web interface system, (2) web server, and (3) Android clients. SWIS: simple web interface system.

The UBESAFE system has two major components (Figure 1):

1. Simple web interface system (SWIS), which allows the health administrator to add, modify, and delete messages and point of interest (POI, known as hot zone)
2. Mobile app developed for Android operating system version 6.0.1 allows the mobile users to receive notifications based on a smart context in three ways:
  - Browsing a hotlink (URL Patrol)
  - Using a risk app
  - Being nearby a hot zone (Localization)

These notifications are any of both health messages provided by a health administrator and user private messages. User can also interact with other functionalities such as the following:

- Evaluate a message with a Likert scale
- Share a message or POI with the web server
- See user stats on the gamification module
- Contact a health professional

### Flux of Work of UBESAFE System

**Figure 2:**
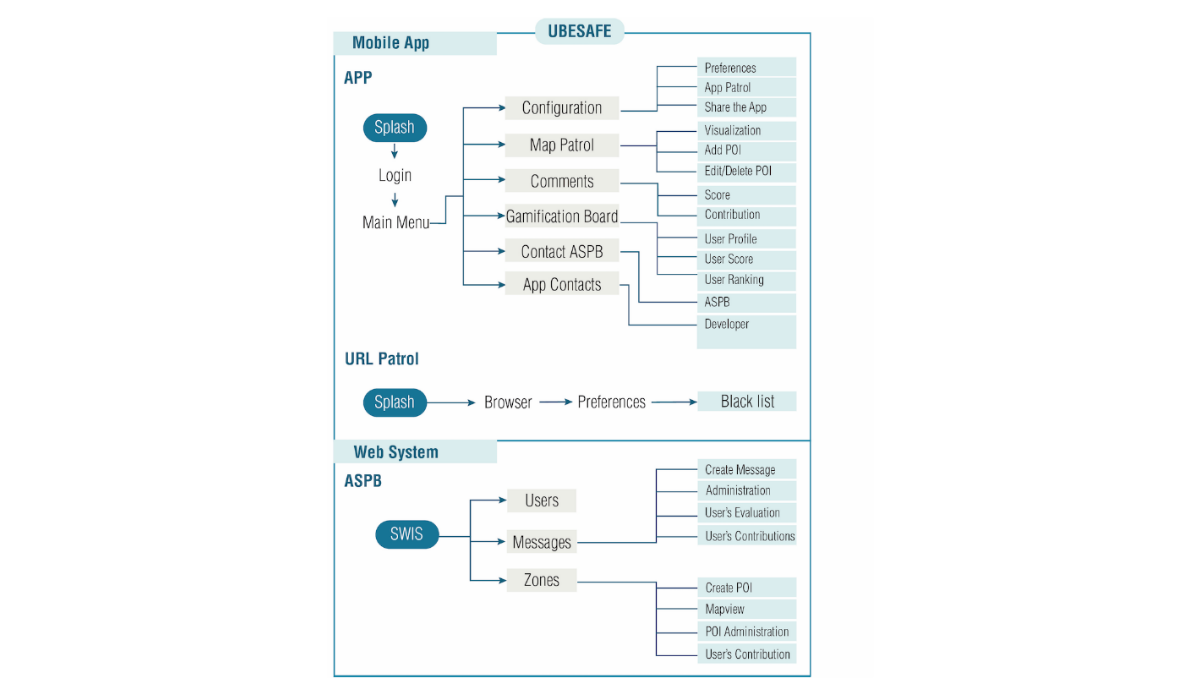
Flux of work of the app. ASPB: public health agency of Barcelona; SWIS: simple web interface system; POI: point of interest.

To understand all functionalities and how the app is structured, the following system is presented (see Figure 2):

1. Mobile app: the mobile app has two main activities:
  - URL patrol, which has all the functionalities of a web browser but with the preventive system incorporated.
  - UBESAFE, which works by detecting the different situations that can make the users aware of their actions through a health message. Thus, the message will be ranked by the users on a Likert scale from 1 to 5, where 1 is the least interesting and 5 is the most interesting.
2. SWIS, through which the health administrator is able to approve, modify, or delete the messages and POIs shared by the users. The system also shows statistics of users’ scoring and most valued messages, number of users, etc.

As can be seen from Figure 1, it is important to note that UBESAFE is an app supervised by health professionals, who can benefit from the knowledge taken from the app to improve their prevention campaigns and also ensure that the messages in the app will always be true and respectful.

### UBESAFE App

The mobile app UBESAFE has two main activities with their own functionalities:

- First, UBESAFE includes the detection of any of both proximity to specific areas (what we call *hot zones*) and the use of any apps that the user wants to be warned on using it (what we call *risky apps*).
- Second, URL patrol notifies the users when it detects what we call a *hot URL*, that is, a URL that the user has marked as one to be warned when clicking on it.

It is important to note that all health messages are retrieved from a local database. This database is controlled from the SWIS and updated every time that a new message or POI is detected, and the administrator releases a new version of the database.

The functionalities and the flux of work for UBESAFE is presented in Figure 2.

The first time that UBESAFE is run on the mobile device, the mobile users will have to fill in their data and configure the app. The process is performed in three steps. First, once the app is opened by touching the UBESAFE icon, the app will check the user data. Second, a preference list will be shown to fill with their information. Finally, once the users have entered all the information, the app will process in background to sign in the users to the SWIS database and download the messages and POI available from the SWIS to query them locally (this process happens on the login section of the Figure 2). Once the mobile users have completed the information requested by the app, the health administrator will be able to see the users’ data in the users’ section of the SWIS system, as will be shown in SWIS section. The app allows the users to participate in the community sharing data for research purposes, or they can choose to be anonymous.

#### Detecting Risk Apps

For detecting risk apps, the users need to configure the service known as AppPatrol. This service will show the mobile users all the apps installed on their device. The mobile users will select the apps for monitoring and then activate the service. This service, similar to all the services of UBESAFE, works in background, and the mobile users do not need to start it again. It will continuously be monitoring the device until the service is deactivated.

It is important to note that it is the user who decides to add the option to receive a warning on using the app. If the users do not add, they will not get any message on using the app. It is important to note that all the process is within the smartphone, and no data are stored regarding the use of the app or the apps for which the user has added to be warned.

**Figure 3:**
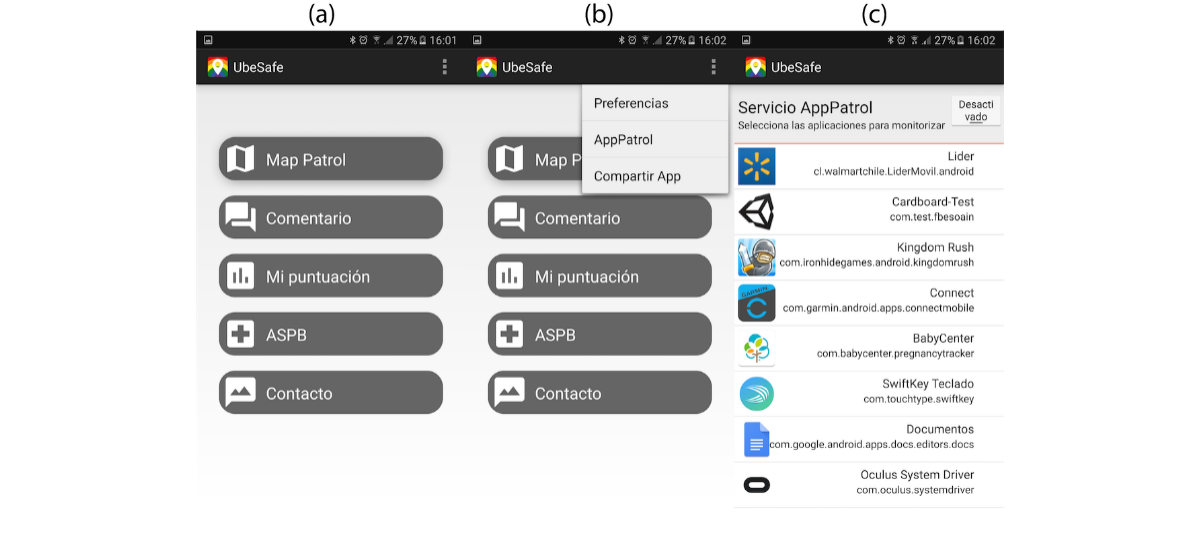
UBESAFE—Android client: setting the AppPatrol service on the app.

Figure 3 shows three states of the app at different times: (1) once the app is opened by touching the UBESAFE icon, (2) the users select in the preference section the AppPatrol settings, and (3) a list with all the icons and name of the installed apps will be shown (this service is off by default).

Once the users activate the service, they can choose the apps they wish to be warned about. Right part of Figure 3 shows the list of installed apps, the user selects apps from the list to monitor. This action is performed by doing a long press on the list (according to the mobile standards, a long action present selection on a list). Thereafter, the user starts the service. This service is always on (algorithms to optimize this service are shown in section Detecting Hot Zones).

#### Detecting Hot Zones

**Figure 4:**
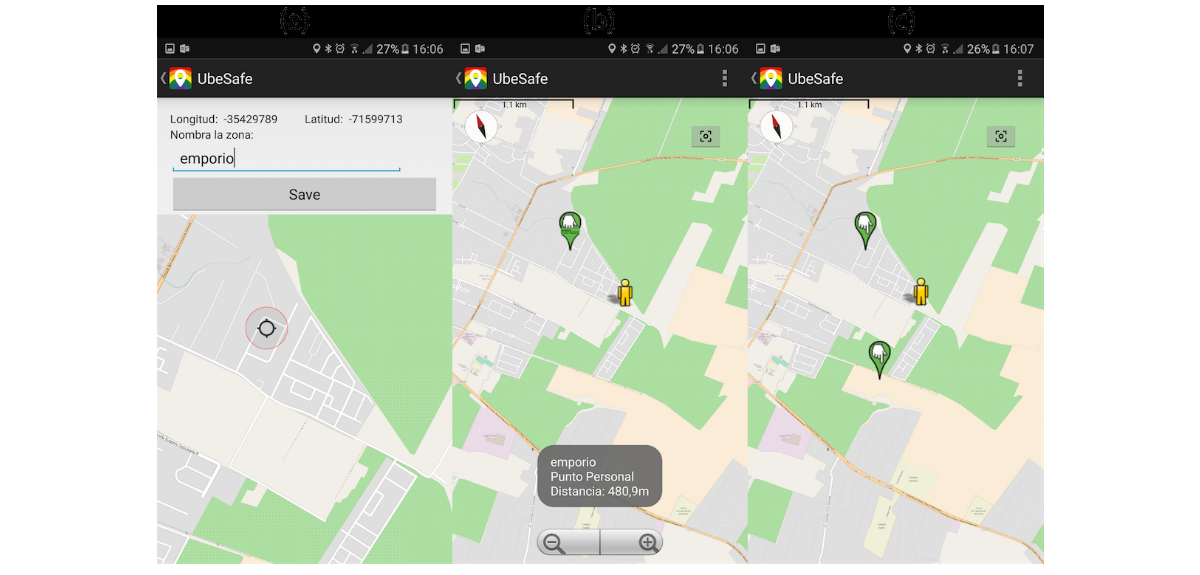
UBESAFE—Android client: adding users’ favorite hot zones for future monitoring of the alert service.

The module to detect hot zones is known as Map Patrol (see Figure 4); it is responsible for sending health messages to users when the app detects the proximity to areas with a high probability of intercourse (hot zones). In addition, the UX has been enhanced with several functionalities:

- To add private POI to the database of the mobile devices.
- To share the POI with the community sending the information to the Information server.
- To delete any POI from the database, allowing the mobile users to choose which hot zones to detect.

When the user has selected the Map Patrol option, the app opens a mapView with the user’s current positions and POI or hot zones nearby. In the configuration section, the mobile users can add POIs and manage a single POI (share it with the community or delete it). It is important to note that UBESAFE allows users to add their own POI, and users themselves decide if they want to share them with the community or not. This option helps to increase the value of the app for users because they can use it to keep their own POI.

Figure 4 shows three states of the app at different times:

- First, the users select adding POI in the Map Patrol settings. In the mapView interface, the users select from a mapView a point by doing a long press on the map. The app automatically will get the latitude and longitude; thereafter, the users must write a name of the POI.
- Second, the app shows the mapView with the recently added POI. If the user touches the POI, then UBESAFE shows information and distance from the user’s current position to the POI.
- Third, on the right, two POIs can be seen nearby the user’s current position.

Besides adding their own POI to the app, mobile users also have the option of sharing the POI with the community by sending the information to the information server, and deleting any POI from the database, allowing the mobile users to choose which hot zones to detect. To do this, (1) the users have to select to manage POI in the Map patrol settings; (2) the app shows a list with the POIs (it is important to note that each POI has an icon to the left that shows the current status of the POI: shared or local); and (3) by doing a long press on the target POI, the mobile users can delete the POI from the local database. In addition, the users can select the POI to share with the community by doing a simple press.

### Managing Health Messages

To improve the functionality and UX related to the health messages, UBESAFE allows the mobile users to score the health message received. The app uses the notification manager service of the Android operating system and presents the message on the notification bar. Moreover, because the app is running in the background all the time, there is no action needed by the users to activate this detection.

**Figure 5:**
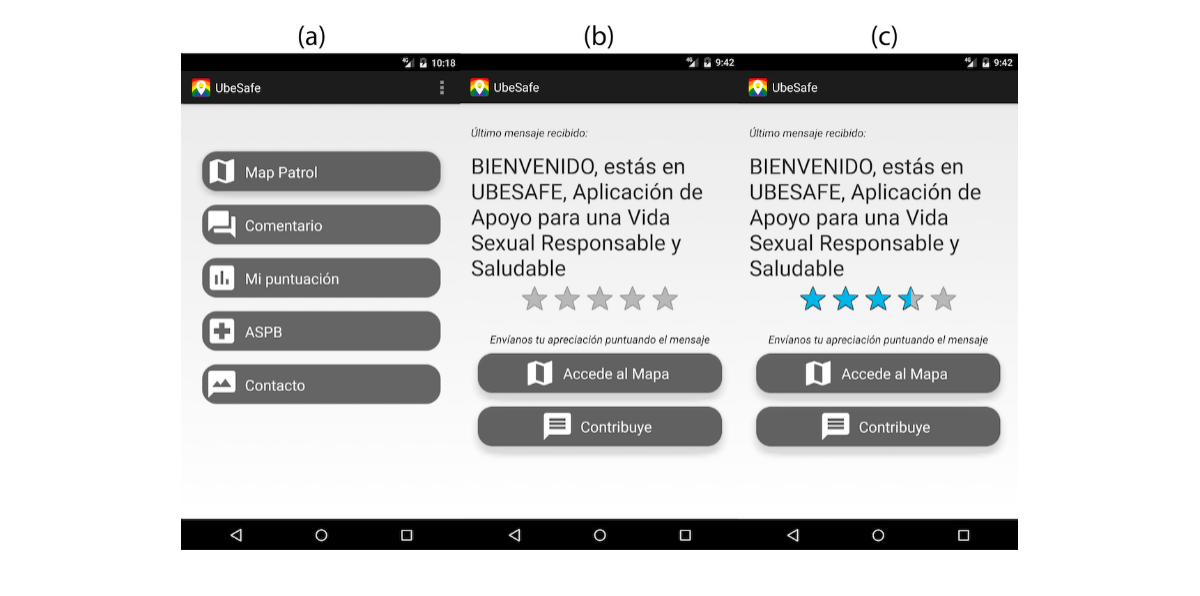
UBESAFE—Android client: primary interface for scoring messages with shortcuts for contribution and mapView.

This interface includes two shortcuts to the map patrol; thus, the mobile users can see their position and all the POIs nearby, which allows the mobile users to add, delete, and upload their own health messages. The mobile users can access this interface from the main menu in *Comments* (see left side of Figure 5) or any time they get a new notification.

When the users open the notification, the app will prompt them to rank the received message out of 5 stars, as it is shown in the center and right side of Figure 5. After the ranking process, the app sends this score to the SWIS where the health administrator can see the average and the highest scored message.

Mobile users can add their own messages because they can have private messages for their consideration. They can also, if they wish, upload, and share their private messages with the community. When the users want to share a message, it will be uploaded to the information server where the health administrator can review it through the SWIS. This revision could modify the original message if needed. Thereafter, the message will be added to the system main database. Thus, users can contribute and add the messages that they think can be more helpful to result in a behavioral change.

To share a message with the community, the users must perform the following procedure: (1) the app shows a list with the messages, and the mobile users can add or upload a message to the information server; (2) the users, by doing a simple press, select the message and then select the sync symbol; and (3) the message has changed the icon from the left, showing that the private message is updated to the web server.

#### Gamification Scoreboard

UBESAFE has a scoreboard with the most valued health messages and users’ ranking. Every time that mobile users share a POI or health message, they get an amount of points of experience in exchange: the amount of experience awarded is related to the number of actions that the users have performed in the system; the more messages or POIs they share with the community, the more experience they get. Depending on the amount of experience, mobile users will get a medal that reflects their rank in the system. The contribution will also be presented on a scoreboard, enhancing the experience with the system and promoting the sharing and contributing of POI and messages to the UBESAFE system.

In the gamification section of the app, first, the app shows the main menu, where the users select the punctuation; and second, the users can see the number of shared contributions (messages and POI). They can also see a bar of experience and the current medal; the image from the right shows how the users have increased their experience in the bar. This happens because the mobile users have shared more messages and POIs.

The use of experience points, medals, and ranking in UBESAFE is an example of gamification because it uses these elements to motivate users to participate more actively in the community. It promotes a sense of competition between the users and allows them to see their accomplishments.

It is important to note that gamification is within the part of prevention and has two extra effects: (1) users get involved in the preventive messages, and messages can be better tuned for target users preferences; and (2) by sharing POIs and messages, health service can improve the design of prevention campaigns by the language used, as well as by the places in which to launch campaigns.

#### URL Patrol UBESAFE

URL Patrol is part of the UBESAFE app, but it could be run independently as a web browser because it allows users to navigate on the internet. They can open the URL patrol preferences, where they can add or delete any website that users want to be warned about when getting into it. The kind of URLs that one can expect to find here are those related to contact apps or contact websites. The app comes with a preloaded list of websites such as Grindr [28], Manhunt [29], Tinder [24,30].

When the users navigate on a website that is on the list, the system will detect that action and will notify the mobile user with a health message. It is important to mention that URL Patrol is not another mobile app, but part of UBESAFE and can be run independently for UX purposes. Mobile users can configure URL patrol as their default web client and use it to navigate on the internet as part of the detection and prevention system.

### Simple Web Information System

**Figure 6:**
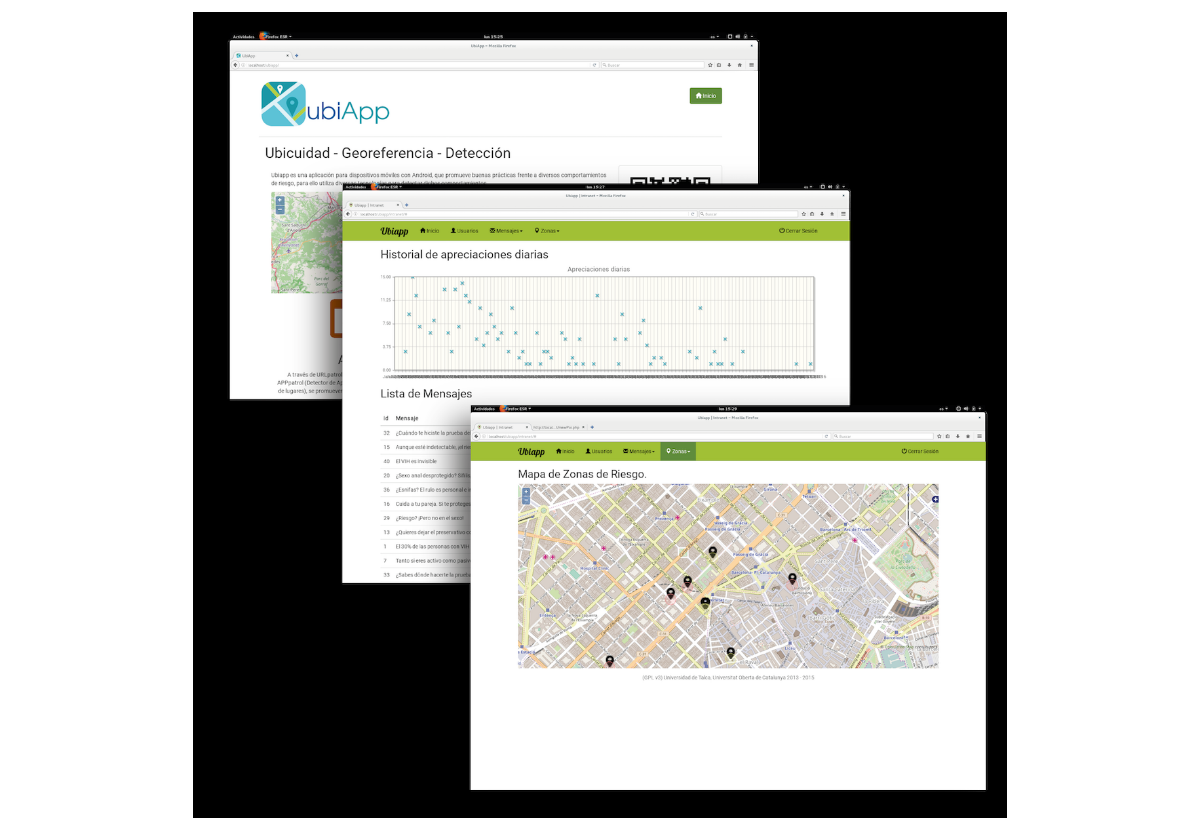
UBESAFE—web client: web interface where the health professional can access and manipulate all the data related to the UBESAFE system.

The SWIS is the interface that allows a health professional to review and check the messages and POIs shared by the mobile users. This interface is important because it is part of a workflow that is controlled by a health professional. The workflow secures the information and validates the messages that will be sent to the mobile users. Figure 6 represents the SWIS with their modules and functionalities.

Once the health administrator gets into the SWIS, the following modules will be presented:

- Users: information about the users (user name, nationality, age, and UID, the internal code that identifies the mobile devices where the UBESAFE app was installed).
- Messages: here the health administrator will be able to create, manage, and delete messages from the system. This will also include the messages shared by the users.
  - Create message: the administrator can add a health message to the system. The message can be in three languages (English, Spanish, and Catalan). If the administrator does not include the message in one of the available languages, the message will not appear in that language.
  - Administration: the administrator can update or delete a specific message. This option is used to modify mobile users’ contributions or refine a proposed health message.
  - User’s evaluation: in this section, the administrator can see the frequency of scored messages by the mobile users per day (graph). Moreover, a list is shown ordered by the average scoring of the system for each message, and the frequency of the scoring by the users per day.
  - User’s contribution: a list with the messages shared by the users is shown. The administrator can update the message, translate it, and approve or delete it. Once the message is approved, it is considered for the next update of the database and shared to the users’ community.
- Zones: here, the health administrator will be able to create, manage, and delete hot zones from the system. This will also include the hot zones and POIs shared by the users.
  - Create POI: the administrator, through positioning a POI into the map interface, is able to add a hot zone to the database.
  - mapView: here the hot zones are shown. Thus, the administrator can have a global and geographic perspective of the data that are stored into the system.
  - POI administration: the administrator can see, approve, or delete POIs in the system. The interface provides the latitude and longitude information and also can show the point on a map, by using Google Maps interface.
  - User’s contribution: a list with the POIs contributed by the users is shown. The administrator can see, approve, or delete every single POI.

See Figure 2 for flux of work of the SWIS.

### Optimization and Key Points

Optimization is a key factor for mobile devices because the main power source (the battery) is limited. The resources that spend more battery on a mobile device are the screen, GPS, long processing times, and an internet connection. Taking this into consideration, all the algorithms were optimized to provide the maximum efficiency in the use of resources.

Optimization and refining have been done during the whole process of software development, considering all the apps developed. In UBESAFE, *AlarmManager* and *broadcaster receiver* allow the app to control the different states of the mobile device during its uptime.

For the alert service, the following states were considered to save battery.

1. It is detected when the mobile device is connected to a USB cable. The connection could be done for two purposes:
  - To charge the mobile device
  - To connect to a computer

In both cases, the mobile device is generally not being used outside, and therefore, there is no need to use the detection of hot zones.

1. The algorithm was enhanced for localization, polling the GPS less than the alert service of the other mobile apps with high accuracy.
2. The update service is not running all the time. It is responsible for retrieving data from the information server every other day because the versions of data are expected to change in a period of days.

For the risk service, the following premise was considered to save battery. Every time that the users are using a contact app on their smartphones, they are using the device. Therefore, the app with the risk services will only be detected when the screen is on. Otherwise, it is assumed that the smartphone is off or in a standby mode. Hence, the current state of the device is checked, using the *PowerManager* of Android application programming interface. Thus, it is possible to infer what the users are doing with the device, starting the risk service when it is necessary rather than all the time.

Finally, the AlarmManager provides access to system-level alarm services. Using the AlarmManager allows an app to schedule tasks that may need to run or repeat beyond the scope of its lifecycle. The Android system tries to batch alarms at similar intervals or times together to preserve battery life. By batching alarms from multiple apps, the system can avoid frequent device waking and networking. Therefore, it saves battery and resources.

### Ethics and Consent Statement

We did not have to ask for ethical approval because at the time of the study, it was not legally necessary to ask for ethical approval for a study where no health-relevant information and personal data were collected from participants because they were already registered in the ASPB, and no personal data are stored within the app. Nevertheless, the users volunteered to become part of the project, received no financial compensation, and could leave the project whenever they wanted. In addition, all the information was shared with them.

### Test the App

As mentioned in the overview, two tests were performed on the app. The first was a functional test with the objective to receive user feedback about technical aspects and usability, whereas the second was a feasibility test to try the UBESAFE system with all of its functionalities. Below, the results are discussed in further detail.

## Results

### Functional Testing Results

The demographic characteristics of the sample are shown in Table 1. Two of the participants are in their 30s and two are older than 40 years. Four of them were Spanish, and one was from Chile.

All the volunteers declared using the mobile device as their primary device for accessing the internet. Moreover, all five of them have an internet plan on their devices. Therefore, they were fully connected the whole time. In this context, they also declared to be constantly aware if the devices have some notification or message. This effect was increased by the ubiquity of the information. Today, a notification on the mobile device is information from a message, text, email, or game notification, among others.

**Table 1:** Sociodemographic characteristics of volunteers.

Age (years)	Occupation	Level of studies	Country of birth
27	Nurse	Undergraduate degree	Spain
42	Interior designer	Undergraduate degree	Spain
30	Medic	Undergraduate degree	Chile
37	Receptionist	Professional degree	Spain
45	Actor	Undergraduate degree	Spain

In addition, all five of the volunteers mentioned that they used the mobile device to chat with friends. Meanwhile, two of them also declared that they use the mobile device to chat with unknown people. In this context, the mobile device was also used to search for information related to health topics such as STIs, HIV, health centers, and sports.

The volunteers used UBESAFE for 2 weeks with the following detections:

- *Execution of some kind of app*: applies in situations where users open apps designed for contacting sexual partners, such as *Manhunt*.
- *Proximity to a geographical zone where sexual contacts often take place*: applies in situations where users enter or are near to what we call a *hot zone*.
- *Detection of a target URL*: applies in situations where users open a target URL.

It is important to note that this version of the app only sends a health message. Once the notification has been displayed, the software can interact with other installed apps, for example, allowing the users to share the notification through email, text message, or social networks. Thus, users have a fully connected experience that can also help to promote prevention among others. When they receive a notification, the users can make an informed decision regarding the possible consequences of their behavior. As a result, the use of this software raises users’ awareness of their actions and encourages them to take steps to limit the spread of STIs.

All of the volunteers declared that they were able to install and configure the app without problems and the app did not compromise the standard functioning of the devices. Regarding the three main functionalities of UBESAFE in this version (detection of hot zones, risk apps, and URL of contact), two of the volunteers mentioned that they received health notification when they were near a hot zone or using a risk app, and three received a health message through the URL detection when they were navigating on the internet.

As part of the discussion and conclusions of the experience of the focus group, Table 2 describes the highlights to be considered: with this first initial testing, it was possible to test the three most important functionalities of detection of the apps and know the users’ perceptions with the aim of enhancing the UX of the system. This test also had a technical tracking of bugs and issues through the Google Play platform for developers. Finally, there is a continuous refinement of the modules of the app as part of the iterative development methodology. All these experiences have increased the value of the product, with more emphasis on the users than on the process.

**Table 2:** Perceptions of volunteers in the functional test.

Volunteers comments	Analysis
I got a lot of messages for the use of one application	This feature was developed on purpose in the first version to see how the mobile users will react to the notifications on demand. Next versions allowed a configuration of the timing of the notifications.
Battery consumption when I use the map	Battery consumption is an issue in all apps that require the constant processing of data. In this case, the use of the mapView consumes energy from two principal sources: localization and screen.
Repetition of the health messages in the different detections	This happens because the experiment had 20 messages for testing purposes. The messages will be presented randomly. A big database of health message is required to avoid repetition.
It is necessary to have more hot zones	Some zones are provided by the health administrator, but there is knowledge that only MSM^a^ know and could be beneficial for the community and future interventions of the public health service.

**^a^**MSM: men who have sex with men.

### Feasibility Testing Context

Four MSM volunteers were enrolled to participate actively in the feasibility evaluation of the UBESAFE system. They were enrolled in Barcelona, a city with an important offer of gay leisure. All the participants declared that they utilized social networks through their smartphones frequently (at least twice a day). They were aged between 27 and 45 years. It is important to consider that the UBESAFE system aims to have a preventive role, making users more conscious of their actions.

### Data Sources and Collection

The data were collected through the smartphones owned by the users. All the data obtained from the system were stored in the database as the foundation of the analysis. Moreover, the data were collected through the SWIS and analyzed concurrently. It is relevant to mention that all the users agreed to share the information for further analysis; setting this option was a requirement to run the app.

Data were collected at three different times: first, through the entry survey; second, through the smartphones; finally, through a final focus group. The objective of the entry survey was to learn more about the target group, regarding their knowledge, habits, and behaviors. The aim of the final focus group was to learn about the UX experience with UBESAFE and collect software suggestions.

The app collects the following three types of data at different times:

1. Profile information: this information is collected the first time that the users run the app. At that moment, the users’ contact information is requested such as user name, email, age, and nationality. In addition, the users must set if they want to share this information with the system or use the system anonymously. With these data, users’ profiles are created on the SWIS that later are related with their scores on the health messages.
2. Perceptions: when UBESAFE detects any of the three detection systems, it will notify the users in the notification bar of the smartphone. The users will be prompted to read the health message and score it on a scale from 1 to 5 (Figure 5).
3. Question for the health administrator: the users were able to send questions to the health administrator in charge (professional of the ASPB).

The health administrator through the SWIS can access to see and review all the data from the system. Several actions can be taken related to the Create, Read, Update, and Delete (CRUD) of messages and POIs on the system. Moreover, the health administrator has a key role in the revision and approval of health data provided by the smartphone users. In addition to the management options, the SWIS shows the list of messages and graphs of perceptions of the users. These features add value to the system because the administrator of the system can see in real time the positive or negative impact of a health message.

### Feasibility Evaluation

In the feasibility evaluation, 1 health professional from the ASPB participated, writing the initial database with 30 messages and responding to the users’ questions. The initial messages were taken from the study presented in the UBIAPP testing experiment, and the questions were responded to within 24 hours. The study had a duration of 30 days; 357 evaluation of health messages were registered in total in the system (2.9 evaluation average per volunteer per day).

The messages come from two sources:

1. Health administrator: the test was begun with 30 messages related to prevention of risky behaviors in MSM. Ten messages were taken from the UBIAPP study. In that study, volunteers were asked to choose the messages they found most and least suitable as preventive messages and were also offered the chance to propose new messages. In addition to this initial list of messages, the ASPB provided 20 additional messages that were reviewed by health professionals of the institution. The messages were displayed randomly by the app.
2. Smartphone users: users are able to share messages and POI with the system. Therefore, a sustainable way was created to maintain the system with new information and encourage users through a gamification system. To keep the primary objectives of the messages, POI, and system, the health administrator must review these data.

The mean of the ratings received during the whole period was 4.60, indicating that the information sent to participants was highly rated in general. A total of 64 messages were registered in the SWIS; therefore, 34 messages were shared by users and added to the 30 original pool of messages. Ten more messages were shared and were not considered appropriate to be added to the pool.

It is important to highlight those messages scored with the highest rating by the participants, considering only those with scores above average plus one standard deviation. Four out of 10 of the highest rated messages were shared by the users rather than the administrator. These messages are shown in Table 3.

Now that the information about the health messages has been seen, it is necessary to turn to the POIs and hot zones. The initial database started with seven hot zones entered by the health administrator. After the test, the database ended up with 16 hot zones. Therefore, 56% (9/16) of the hot zones were contributions of the smartphone users. This information is very important for the health service because it allowed them to identify new zones for launching prevention campaigns.

**Table 3:** Messages sent by the system scored with the highest rating by the participants.

Message	Average scoring	Frequency
When was the last time that you got tested for HIV?	5.0	10
Even if nothing has been detected, 0 risk doesn’t exist!	4.95	10
HIV is invisible	4.91	11
Unprotected anal sex? You can get syphilis, gonorrhea and other STI^a^	4.86	11
Do you snort? The tube is personal and non-transferable	4.80	10
Take care of your partner. If you protect yourself, you protect him	4.79	12
Risk? But not in sex!	4.70	10
Do you want to stop using a condom with your guy? Let’s get tested together	4.70	15
Oral sex has also some risks	4.68	10
Let us be serious against HIV.	4.6	9

^a^STI: sexually transmitted infection.

## Discussion

In this paper, we presented the app UBESAFE, which sends preventive notifications to users when it detects situations such as the activation of particular apps on their smartphones, the access to a specific URL on the internet, or their proximity to areas with a high probability of intercourse (*hot zones*). It also develops community for the users, considering their ideas and knowledge of the hot zones. To provide a sustainable way of getting new data, the main experience was developed with *gamification* concepts. It is important to note that UBESAFE wants to create awareness of each detected situation through health messages, considering privacy and users’ preferences.

Therefore, the app works on four main lines:

- Sending health messages to users when the app detects a hot URL, that is, a website where users can meet or chat with unknown people.
- Sending health messages to users when the app detects the use of a contact app (that we call risk apps such as Manhunt, Tinder, Badoo, or Brenda, among others).
- Sending health messages to users when the app detects the proximity to areas with a high probability of intercourse (hot zone), such as saunas, intercruising zones, etc.
- Allowing the users to make a community sharing messages and POI (hot zones) through the system, enhancing the experience with a gamified scoreboard.

UBESAFE adds new modules and functionalities to the previously developed Android apps Ubiapp [12], Ubinut, and Geonut [19] through its modular architecture. It is a supervised app designed not only to have a health system that helps to keep information within the app respectful and true but also to help these professionals to know their target better.

The app was tested for 30 days. During the trial period, 357 evaluations were received from users, which rated the different messages on a Likert scale of 1 to 5, obtaining an average of nearly 2.9 responses a day per user. The mean of the ratings received during the whole period was 4.60, indicating that the information sent to participants was highly rated in general.

The volunteers highly valued the functionalities related to sharing information and seeing how their peers valued it. They felt they are part of an informed community that helps to improve the knowledge on this matter, enhancing the UX and purpose behind installing and being part of this app. In fact, users increased the pool of messages by more than 100% (34/30) and created 56% (9/16) of the hot zones.

The app shows, on one hand, its value to the target users, and how the gamification can increase adherence to the app, and on the other hand, how it can help the health professionals to know their target users and prevention campaigns better. Thus, UBESAFE is an app that can help in the prevention of HIV and other STIs.

Finally, further work will address the following:

1. Improving the graphic design and UX feedback of the gamified board
2. Implementing a more accurate ontology algorithm to improve the automatic system that sends messages in UBESAFE, making the selected messages closer to those that a health professional would choose in each situation
3. Measuring the impact of UBESAFE as a ubiquitous system for promoting healthy habits through an evaluation methodology, such a longitudinal study.
4. Explore a theoretical perspective from the social psychology of communication to advance an explanation of the effects of STI prevention strategies through mobile devices, using a theory-based technological solution that implies the development of positive attitudes toward the use of condoms and positive sexual health behaviors through an active generation of consequent favorable thoughts (ie, the Elaboration Likelihood Model of persuasion [17]).

## Abbreviations

ASPB: public health agency of Barcelona
MSM: men who have sex with men
POI: point of interest
STI: sexually transmitted infection
SWIS: simple web interface system
UX: user experience
